# Two β-Galactosidases from the Human Isolate *Bifidobacterium breve* DSM 20213: Molecular Cloning and Expression, Biochemical Characterization and Synthesis of Galacto-Oligosaccharides

**DOI:** 10.1371/journal.pone.0104056

**Published:** 2014-08-04

**Authors:** Sheryl Lozel Arreola, Montira Intanon, Jasmina Suljic, Roman Kittl, Ngoc Hung Pham, Paul Kosma, Dietmar Haltrich, Thu-Ha Nguyen

**Affiliations:** 1 Food Biotechnology Laboratory, Department of Food Science and Technology, BOKU - University of Natural Resources and Life Sciences, Vienna, Austria; 2 Institute of Chemistry, University of the Philippines Los Baños, College, Laguna, Philippines; 3 School of Food Biotechnology and Food Technology, Hanoi University of Science and Technology, Hanoi, Vietnam; 4 Division of Organic Chemistry, Department of Chemistry, BOKU - University of Natural Resources and Life Sciences, Vienna, Austria; Universidade Nova de Lisboa, Portugal

## Abstract

Two β-galactosidases, β-gal I and β-gal II, from *Bifidobacterium breve* DSM 20213, which was isolated from the intestine of an infant, were overexpressed in *Escherichia coli* with co-expression of the chaperones GroEL/GroES, purified to electrophoretic homogeneity and biochemically characterized. Both β-gal I and β-gal II belong to glycoside hydrolase family 2 and are homodimers with native molecular masses of 220 and 211 kDa, respectively. The optimum pH and temperature for hydrolysis of the two substrates *o*-nitrophenyl-β-D-galactopyranoside (*o*NPG) and lactose were determined at pH 7.0 and 50°C for β-gal I, and at pH 6.5 and 55°C for β-gal II, respectively. The *k*_cat_/*K*_m_ values for *o*NPG and lactose hydrolysis are 722 and 7.4 mM^−1^s^−1^ for β-gal I, and 543 and 25 mM^−1^s^−1^ for β-gal II. Both β-gal I and β-gal II are only moderately inhibited by their reaction products D-galactose and D-glucose. Both enzymes were found to be very well suited for the production of galacto-oligosaccharides with total GOS yields of 33% and 44% of total sugars obtained with β-gal I and β-gal II, respectively. The predominant transgalactosylation products are β*-*D-Gal*p*-(1→6)-D-Glc (allolactose) and β*-*D-Gal*p-*(1→3)-D-Lac, accounting together for more than 75% and 65% of the GOS formed by transgalactosylation by β-gal I and β-gal II, respectively, indicating that both enzymes have a propensity to synthesize β*-*(1→6) and β*-*(1→3)-linked GOS. The resulting GOS mixtures contained relatively high fractions of allolactose, which results from the fact that glucose is a far better acceptor for galactosyl transfer than galactose and lactose, and intramolecular transgalactosylation contributes significantly to the formation of this disaccharide.

## Introduction

The colonic microbiota is composed of more than 400 different species, some of which have been related to health and well-being of the host [1]. In practice, the beneficial bacteria that serve as main targets to be increased in number and/or activity by different approaches are bifidobacteria and lactobacilli [2]. Members of the genus *Bifidobacterium* are one of the most common organisms found in the human gastro-intestinal tract [3], [4]. These species are considered to be important in maintaining human health as they contribute to carbohydrate fermentations in the colon, and their diversity and number provide a marker for the stability of the human intestinal microflora [5]. Bifidobacteria thus play an important role in the eco-physiology of the colonic microbiota, although their population sizes and species composition vary among different groups of human population. The major *Bifidobacterium* species found in the adult microflora are *Bifidobacterium adolescentis* and *B. longum* while *B. infantis* and *B. breve* are the predominant bifidobacteria in infant intestinal tracts [4], [6]. Activity of these bacteria has been linked to health effects such as increased resistance to infection, stimulation of the immune system activity, protection against cancer, or other prophylactic and therapeutic benefits. Bifidobacteria are also known to excrete a range of water-soluble vitamins such as folate, nicotinic acid, thiamine, pyridoxine, and vitamin B12 [7].

Prebiotic oligosaccharides can serve as fermentable substrates for certain members of the gut microbiota and have been found to modulate the colonic flora by selective stimulation of the beneficial bacteria as well as inhibition of ‘undesirable’ bacteria [7]–[9]. Galacto-oligosaccharides (GOS) are the products of transgalactosylation reactions catalyzed by β-galactosidases when using lactose as the substrate and can include various trisaccharides, higher oligosaccharides as well as non-lactose disaccharides. GOS are non-digestible carbohydrates meeting the criteria of ‘prebiotics’ [10], and have attracted increasing attention because of the presence of structurally related oligosaccharides together with different complex structures in human breast milk. Therefore the use of GOS in infant formula is nowadays of great interest [11]–[13].

β-Galactosidases (β-gal; EC 3.2.1.23) catalyze the hydrolysis and transgalactosylation of β-D-galactopyranosides (such as lactose) [14]–[16] and are found widespread in nature. They catalyze the cleavage of lactose (or related compounds) in hydrolysis mode. An attractive biocatalytic application is found in the transgalactosylation potential of these enzymes, which is based on their catalytic mechanism [14], [17]. The use of lactic acid bacteria (LAB) and bifidobacteria as sources of β-galactosidases may offer substantial potential for the production of GOS [18], and recent years have seen a significant increase in studies dealing with their biochemical properties or their ability to produce GOS in biocatalytic processes [19]–[34]. *Bifidobacterium breve* DSM 20213 is an isolate from the infant gut. The possibility of rationally targeting prebiotics to specific groups of bacteria such as certain known and approved probiotic strains is a promising prospect. One potential approach to this end is the use of enzymes, such as a β-galactosidase obtained from a probiotic strain, for the synthesis of oligosaccharides [35]. The two β-galactosidases from *B. breve* DSM 20213 selected for this work, β-gal I and β-gal II, are encoded by the corresponding *lacZ* genes (NCBI Reference No. EFE90149.1; EFE88654.1) and belong to glycoside hydrolase family 2 (GH2 family). β-Galactosidases of the GH2 family generally receive more attention in terms of transgalactosylation activity since they may have a high propensity to catalyze this reaction.

In this paper, we describe the cloning of two β-galactosidases from *Bifidobacterium breve* DSM 20213 and their expression in *Escherichia coli*. Furthermore, biochemical properties of these enzymes and their potential to produce GOS are also presented.

## Materials and Methods

### Chemicals and vectors

All chemicals and enzymes were purchased from Sigma (St. Louis, MO, USA) unless stated otherwise and were of the highest quality available. The test kit for the determination of D-glucose was obtained from Megazyme (Wicklow, Ireland). All restriction enzymes, T4 DNA ligase, and corresponding buffers were from Fermentas (Vilnius, Lithunia). The plasmid pET-21a (+) was from Novagen (Darmstadt, Germany) and the plasmid pGRO7 encoding the chaperones GroEL and GroES was from TAKARA (Shiga, Japan). Galacto-oligosaccharide standards of β*-*D-Gal*p*-(1→3)-D-Glc, β*-*D-Gal*p*-(1→6)-D-Glc, β*-*D-Gal*p-*(1→3)-D-Gal, β*-*D-Gal*p-*(1→4)-D-Gal, β*-*D-Gal*p-*(1→6)-D-Gal, β*-*D-Gal*p-*(1→3)-D-Lac, β*-*D-Gal*p-*(1→4)-D-Lac, β*-*D-Gal*p-*(1→6)-D-Lac were purchased from Carbosynth (Berkshire, UK).

### Bacterial strains and culture conditions

*B. breve* DSM 20213, an infant isolate, was obtained from the German Collection of Microorganisms and Cell Cultures (DSMZ, Braunschweig, Germany). The strain was grown anaerobically at 37°C in MRS medium [36]. *Escherichia coli* DH5α (New England Biolabs, Frankfurt am Main, Germany) was used in the transformation experiments involving the subcloning of the DNA fragments. *Escherichia coli* T7 express (Novagen) was used as expression host for the vectors carrying the target DNA fragment encoding β-galactosidases.

### Construction of β-galactosidase expression vectors

The β-gal I (NCBI Reference No. EFE90149.1) and β-gal II gene (NCBI Reference No. EFE88654.1) were amplified using proof-reading Phusion polymerase with the primer pairs 5BbBG1Nde1 (5′-AATACATATGCAAGGAAAGGCGAAAACC-3′), 3BbBG1Not1 (5′-ATAGCGGCCGCGATTAGTTCGAGTGTCACATCC-3′) and 5BbBG2Nde1 (5′-AATACATATGAACACAACCGACGATCAG-3′), 3BbBG2Not1 (5′-ATAGCGGCCGCGATGAGTTCGAGGTTCACGTC-3′), respectively. The forward primers contain *Nde*I and the reverse primers include *Not*I recognition sites (underlined). The template for the PCR reaction was obtained from cells scratched from an MRS agar plate and suspended in the PCR mix. The initial denaturation step at 98°C for 3 min was follow by 30 cycles of denaturation at 95°C for 30 s, annealing at 60°C for 30 s and extension at 72°C for 2 min, followed by a final extension step at 72°C for 5 min. The amplified genes were digested with the corresponding restriction enzymes. Subsequently, the gene fragments were ligated into the pET-21a(+) vector without the natural stop codon and in frame with the C-terminal His_6_-tag sequence on the vector, and transformed into *E. coli* DH5α cells. The resulting expression vectors β-gal I and β-gal II were transformed into two different hosts, *E. coli* T7 Express and *E. coli* T7 Express carrying the plasmid pGRO7 (*E. coli* T7 Express GRO), for comparison of the expression levels. The correct nucleotide sequences were confirmed by sequencing (VBC-Biotech, Vienna, Austria). The basis local alignment search tool (BLAST) from the National Center for Biotechnology Information BLAST website was used for database searches. The comparison of β-galactosidases from *B. breve* with homologous proteins was carried out using the program ClustalW2 (version 2.0) [37].

### Heterologous expression of β-galactosidases

The expression levels of β*-*gal I and β*-*gal II with and without co-expression of the chaperones GroEL and GroES were compared. To this end, all cultures were grown at 37°C in 250 mL of MagicMedia (Invitrogen, Carlsbad, CA, USA) until an optical density at OD_600 nm_ of 0.6 was reached. MagicMedia promotes high expression levels of T7-regulated genes without the need of adding inducing reagent such as IPTG. The co-expression of the chaperons was induced with 1 mg mL^−1^ L-arabinose. The cultures were incubated further at 20°C overnight. The cells were harvested by centrifugation (6,000×*g*, 30 min, 4°C), washed twice with 50 mM sodium phosphate buffer, pH 6.5, and disrupted using a French press (AMINCO, Silver Spring, MD). The resulting homogenate was centrifuged at 25,000×*g* for 30 min at 4°C to remove the cell debris. The crude extracts were tested for protein concentration and β-galactosidase activity using the standard assay.

Subsequently, the expression of β*-*gal I and β*-*gal II with co-expression of the chaperones was studied further. In this investigation, LB medium was used instead of MagicMedia. Different induction conditions were compared by varying the concentrations of isopropyl-β-D-thiogalactopyranoside (IPTG) in LB medium. *E. coli* T7 express GRO cells carrying β-gal I and β-gal II plasmids, respectively, were grown at 37°C in 100 mL of LB medium containing 100 µg mL^−1^ ampicillin and 1 mg mL^−1^ L-arabinose for chaperone induction until an optical density at OD_600 nm_ of ∼0.8 was reached. IPTG was added to the culture medium in various final concentrations (0.1, 0.5, 1.0 mM), and the cultures were incubated at 18°C for 16 h. The cultures were harvested, washed twice and resuspended in 1 mL of 50 mM sodium phosphate buffer, pH 6.5. Cells were disrupted in a bead beating homogenizer using 0.5 g of glass bead (Precellys 24 Technology; PEQLAB, Germany), which is practical for small sample volumes used here. The crude extracts obtained after centrifugation (16,000×*g* for 20 min at 4°C) were tested for β-galactosidase activity using the standard enzyme assay and protein concentrations.

### Cultivation and purification of recombinant β-galactosidases

*E. coli* T7 express GRO cells carrying the plasmids β*-*gal I and β*-*gal II, respectively, were grown at 37°C in 1 L LB medium containing 100 µg mL^−1^ ampicillin, 20 µg mL^−1^ chloramphenicol and 1 mg mL^−1^ L-arabinose until OD_600 nm_ of 0.8 was reached. IPTG (0.5 mM for β-gal I and 1 mM for β-gal II) was added to the medium and the cultures were incubated further at 18°C for 16 h. The cultures were then harvested, washed twice with 50 mM sodium phosphate buffer, pH 6.5 and disrupted by using a French press. Cell debris was removed by centrifugation (25,000×*g*, 30 min, 4°C) and the lysate (crude extract) was loaded on a 15 mL Ni-immobilized metal ion affinity chromatography (IMAC) column (GE Healthcare, Uppsala, Sweden) that was pre-equilibrated with buffer A (20 mM phosphate buffer, 20 mM imidazole, 500 mM NaCl, pH 6.5). The His-tagged protein was eluted at a rate of 1 mL min^−1^ with a 150 mL linear gradient from 0 to 100% buffer B (20 mM sodium phosphate buffer, 500 mM imidazole, 500 mM NaCl, pH 6.5). Active fractions were pooled, desalted and concentrated by ultrafiltration using an Amicon Ultra centrifugal filter unit (Millipore, MA, USA) with a 30 kDa cut-off membrane. Purified enzymes were stored in 50 mM sodium phosphate buffer, pH 6.5 at 4°C for further analysis.

### Gel electrophoresis analysis

The purity and the molecular mass of β-galactosidases were determined by SDS-PAGE. The enzymes were diluted to 1 mg protein mL^−1^ and incubated with 2×Laemmli buffer at 90°C for 5 min. Protein bands were visualized by staining with Bio-safe Coomassie (Bio-Rad). Unstained Precision plus Protein Standard (Bio-Rad) was used for mass determination.

### Size exclusion chromatography - Multi-angle laser light scattering analysis

Size exclusion chromatography (SEC) was performed with a Superdex S200 10/300 GL column (GE Healthcare) equilibrated in a buffer containing 20 mM Tris (pH 7.5), 150 mM NaCl, 1 mM β-mercaptoethanol and 5 mM EDTA. The sample separations were performed at room temperature with a flow rate of 0.5 mL min^−1^. The samples (50 µL) were injected as indicated at a concentration of 2.5 mg mL^−1^. On-line multi-angle laser light scattering analysis (MALLS) detection was performed with a miniDawn Treos detector (Wyatt Technology, Santa Barbara, CA) using a laser emitting at 690 nm. The protein concentration was measured on-line by refractive index measurement using a Shodex RI-101 instrument (Showa Denko, Munich, Germany). Analysis of the data was performed with the ASTRA software (Wyatt Technology).

### β-Galactosidase assays

The measurement of β-galactosidase activity using *o*-nitrophenyl-β-D-galactopyranoside (*o*NPG) or lactose as the substrates was carried out as previously described [26]. Reactions were performed in 50 mM sodium phosphate buffer (pH 6.5) at 30°C, and the release of *o*-nitrophenol (*o*NP) was measured by determining the absorbance at 420 nm. One unit of *o*NPG activity was defined as the amount of enzyme releasing 1 µmol of *o*NP per minute under the described conditions. When lactose was used as the substrate, reactions were performed in 50 mM sodium phosphate buffer, pH 6.5, at 30°C. After stopping the reaction, the release of D-glucose was determined using the test kit from Megazyme, in which D-glucose was assessed colorimetrically using the GOD/POD (glucose oxidase/peroxidase) assay. One unit of lactase activity was defined as the amount of enzyme releasing 1 µmol of D-glucose per minute under the given conditions. Protein concentrations were determined by the method of Bradford [38] using bovine serum albumin as the standard.

### Steady-state kinetic measurement

All steady-state kinetic measurements were obtained at 30°C using *o*NPG and lactose as the substrates in 50 mM sodium phosphate buffer, pH 6.5, with concentrations ranging from 0.5 to 22 mM for *o*NPG and from 1 to 600 mM for lactose, respectively. The inhibition of *o*NPG hydrolysis by D-galactose and D-glucose as well as that of lactose hydrolysis by D-galactose using various concentrations of the inhibitors (ranging from 0 to 200 mM) was investigated as well. The kinetic parameters and inhibition constants were calculated by nonlinear regression, and the observed data were fit to the Henri-Michaelis-Menten equation (SigmaPlot, SPSS Inc., Chicago, IL).

### pH and temperature dependency of activity and stability

The pH dependency of the recombinant enzymes was evaluated by the standard assay with 22 mM *o*NPG in the pH range of 3–10 using Briton-Robinson buffer (20 mM acetic acid, 20 mM phosphoric acid, and 20 mM boric acid titrated with 1 M NaOH to the desired pH). To evaluate the pH stability of β-gal I and β-gal II, the enzyme samples were incubated at various pH values using Britton-Robinson buffers at 37°C and the remaining activity was measured at time intervals with *o*NPG as substrate. The temperature optima for hydrolytic activity of β-gal I and β-gal II with both substrates lactose and *o*NPG were determined at 20–90°C and measured at optimum pH of each enzyme. The thermostability was evaluated by incubating the pure enzyme in 50 mM sodium phosphate buffer (pH 6.5) at several temperatures. The residual activities were measured regularly with *o*NPG as substrate. Inactivation constants (*k*_in_) were calculated using first-order rate equations and half-life time of activities (τ_1/2_) were calculated based on these *k*_in_ values.

### Differential Scanning Calorimetery (DSC)

DSC measurements were performed using a MicroCal VP-DSC System (GE Healthcare) controlled by the VP-viewer program and equipped with a 0.137-mL cell. Studies were made with 1 mg mL^−1^ protein samples in 50 mM phosphate buffer (pH 6.5). Samples were analyzed using a programmed heating scan rate of 60°C h^−1^ in the range of 33–80°C. For baseline correction, a buffer blank was scanned in the second chamber and subtracted. Data analysis was performed with the MicroCal Origin software (GE Healthcare) and experimental data points were fitted to an MN2-State Model.

### Substrate specificity

Substrate specificity of the recombinant enzymes was determined using various structurally related chromogenic substrates under standard assay conditions as described for *o*NPG. The chromogenic substrates tested were 2-nitrophenyl-β-D-glucopyranoside, 4-nitrophenyl-β-D-mannopyranoside, 4-nitrophenyl-α-D-galactopyranoside, and 4-nitrophenyl-α-D-glucopyranoside at substrate concentration of 22 mM.

Substrate affinities of the recombinant enzymes towards some galactosides were also evaluated. An appropriate amount of each enzyme was incubated with ∼3 mM of each galactoside (lactose, β*-*D-Gal*p*-(1→3)-D-Glc, β*-*D-Gal*p*-(1→6)-D-Glc, β*-*D-Gal*p-*(1→3)-D-Gal, β*-*D-Gal*p-*(1→4)-D-Gal, β*-*D-Gal*p-*(1→6)-D-Gal, β*-*D-Gal*p-*(1→3)-D-Lac, β*-*D-Gal*p-*(1→4)-D-Lac, β*-*D-Gal*p-*(1→6)-D-Lac) at 30°C in 50 mM sodium phosphate buffer, pH 6.5. Samples were taken after 30 and 60 min and reactions were stopped by incubation at 95°C for 5 min. The relative activities of the recombinant enzymes towards each galactoside were determined considering the percentage of the hydrolysis (or conversion) of each galactoside under similar reaction conditions.

### Galacto-oligosaccharides synthesis and analysis

Discontinuous conversion reactions were carried out at 30°C to determine the transgalactosylation reaction of the recombinant β-galactosidases from *B. breve.* The substrate lactose solution (200 g L^−1^) was prepared in 50 mM sodium phosphate buffer containing 1 mM Mg^2+^. Agitation was applied at 300 rpm with a thermomixer (Eppendorf, Hamburg, Germany). Samples were taken at certain time intervals to determine the residual activities and the carbohydrate contents in the reaction mixtures by high performance anion exchange chromatography with pulsed amperometric detection (HPAEC-PAD). HPAEC−PAD analysis was carried out on a Dionex DX-500 system as previously described in detail [27].

### Statistical Analysis

All experiments and measurements were performed at least in duplicate, and the data are given as the mean±standard deviation when appropriate. The standard deviation was always less than 5%.

## Results

### Expression and purification of recombinant β-galactosidases from B. breve

The β-gal I and β*-*gal II genes were cloned into pET-21a (+). Comparison of amino acid sequences deduced from these two *lacZ* genes revealed 57% of sequence homology. The resulting expression vectors were then transformed into *E. coli* T7 express cells and T7 express cells carrying the plasmid pGRO7. The resulting clones were cultivated under inducing conditions in MagicMedia to compare the expression yields with and without chaperone co-expression. β-Gal I and β-gal II expressed in the strains with chaperones showed a 30- and 14-fold increase in activity compared to the activity obtained from the strains without chaperones, respectively (Table 1). When using these conditions, 193 kU per liter of fermentation broth with a specific activity of 159 U mg^−1^ of β-gal I and 36 kU per liter of fermentation broth with a specific activity of 31 U mg^−1^ of β-gal II were obtained.

**Table 1:** β-Galactosidase activities in cell-free extracts of recombinant *E. coli* expressing *B. breve* β-gal I or β-gal II with and without coexpression of chaperones^a^.

Enzyme	Volumetric activity (kU L^−1^ fermentation broth)^b^		Specific activity (U mg^−1^ protein)		Expression factor^c^ (fold)
		with chaperones		with chaperones	
*β*-gal I	6.4	193.2	1.8	159.0	30.2
*β*-gal II	2.6	36.5	2.5	31.4	14.0

a Values are the mean of two cultivations.

b *o*NPG was used to determine enzyme activity.

c The expression factors are calculated as the ratios of the volumetric β-galactosidase activities obtained from the expressions with chaperones and without chaperones.

The expression levels of both enzymes increased even further when LB medium was used instead of MagicMedia and gene expression was induced using IPTG. The highest yields were obtained when 0.5 mM IPTG was used for induction of β-gal I (683 kU per L of medium with a specific activity of 142 U mg^−1^), and when using 1.0 mM IPTG for β-gal II expression (169 kU per L of medium with a specific activity of 34 U mg^−1^). Both enzymes were subsequently purified with a single-step procedure using an IMAC column. The specific activities of the purified enzymes were 461 U mg^−1^ of protein for β-gal I and 196 U mg^−1^ of protein for β-gal II when using the standard *o*NPG assay. The purification procedure yielded a homogenous β-gal I and β-gal II preparation as judged by SDS-PAGE gel (Figure S1).

### Gel electrophoresis analysis

Both recombinant β-galactosidases from *B. breve* showed molecular masses of approximately 120 kDa as judged by SDS-PAGE in comparison with reference proteins (Figure S1). Molecular masses of 116,127 and 116,594 Da were calculated for β-gal I and β-gal II, respectively, based on their DNA sequences. Size exclusion chromatography in combination with online multi-angle laser light scattering (SEC-MALLS) analysis revealed that the native molecular masses of β-gal I and β-gal II are 220 and 211 kDa, respectively. Therefore, it can be concluded that both enzymes are dimers and it is likely that they are homodimers consisting of two identical subunits as has been shown for other oligomeric β*-*galactosidases of the LacZ type [39], [40].

### Kinetic parameters

The steady-state kinetic constants and the inhibition constants of *B. breve* β*-*galactosidases determined for the hydrolysis of lactose and *o*NPG are summarized in Table 2. The *k*_cat_ values were calculated on the basis of the theoretical *v*_max_ values experimentally determined by nonlinear regression and using a molecular mass of 116 kDa for the catalytically active subunit. β-Gal I and β-gal II are not inhibited by their substrates, which are *o*NPG in concentrations of up to 25 mM or lactose in concentrations of up to 600 mM, as it is evident from the Michaelis-Menten plots (not shown). The end product D-galactose was found to competitively inhibit the hydrolysis of lactose by both enzymes. This inhibition, however, is only moderate as is obvious from the ratio of the Michaelis constant for lactose and the inhibition constant for D-galactose, which were calculated for both enzymes (β-gal I, *K*_i,Gal_/*K_m,_*_Lac_ = 1.8; β-gal II, *K*_i,Gal_/*K_m,_*_Lac_ = 3.6). D-Galactose was also found to be a competitive inhibitor against *o*NPG with inhibition constants of 15 mM for β-gal I and 34 mM for β-gal II. Based on the ratio of *K*_i_ to *K*_m_ this inhibition is even less pronounced (β-gal I, *K*_i,Gal_/*K*_m,*o*NPG_ = 11.5; β-gal II, *K*_i,Gal_/*K*_m,*o*NPG_ = 50.7). *o*NPG was also used as the substrate for studying inhibition by the second end product, D-glucose. Again, glucose is a competitive inhibitor of both enzymes, but this inhibiting effect is only moderate (β-gal I, *K*_i,Glc_/*K*_m,*o*NPG_ = 92; β-gal II, *K*_i,Glc_/*K*_m,*o*NPG_ = 55).

**Table 2:** Kinetic parameters of two recombinant β*-*galactosidases (β-gal I and β-gal II) from *B. breve* for the hydrolysis of lactose and *o*-nitrophenyl β-D-galactopyranoside (*o*NPG).

Substrate	Method for determination of enzyme activity	Kinetic parameter	β*-*gal I	β-gal II
Lactose	release of D-Glc	*v*_max,Lac_ (*µ*mol min^−1 ^mg^−1^)	59±2	97±5
		*K*_m,Lac_	15.3±3.2	7.5±0.9
		*k*_cat_ (s^−1^)	114±4	188±10
		*k*_cat_/*K*_m_ (mM^−1 ^s^−1^)	7.4±1.9	25±4
		*K*_i,Gal_	28±9	27±6
*o*NPG	release of *o*NP	*v*_max,*o*NP_ (µmol min^−1^ mg^−1^)	486±9	188±3
		*K*_m,*o*NP_	1.3±0.1	0.67±0.07
		*k*_cat_ (s^−1^)	939±7	364±6
		*k*_cat_/*K*_m_ (mM^−1 ^s^−1^)	722±66	543±65
		*K*_i,Gal_	15±3	34±5
		*K*_i,Glc_	120±31	37±4

Data given are the mean of two independent experiments ± standard error.

### Effects of temperature and pH on enzyme activity and stability

**Figure 1:**
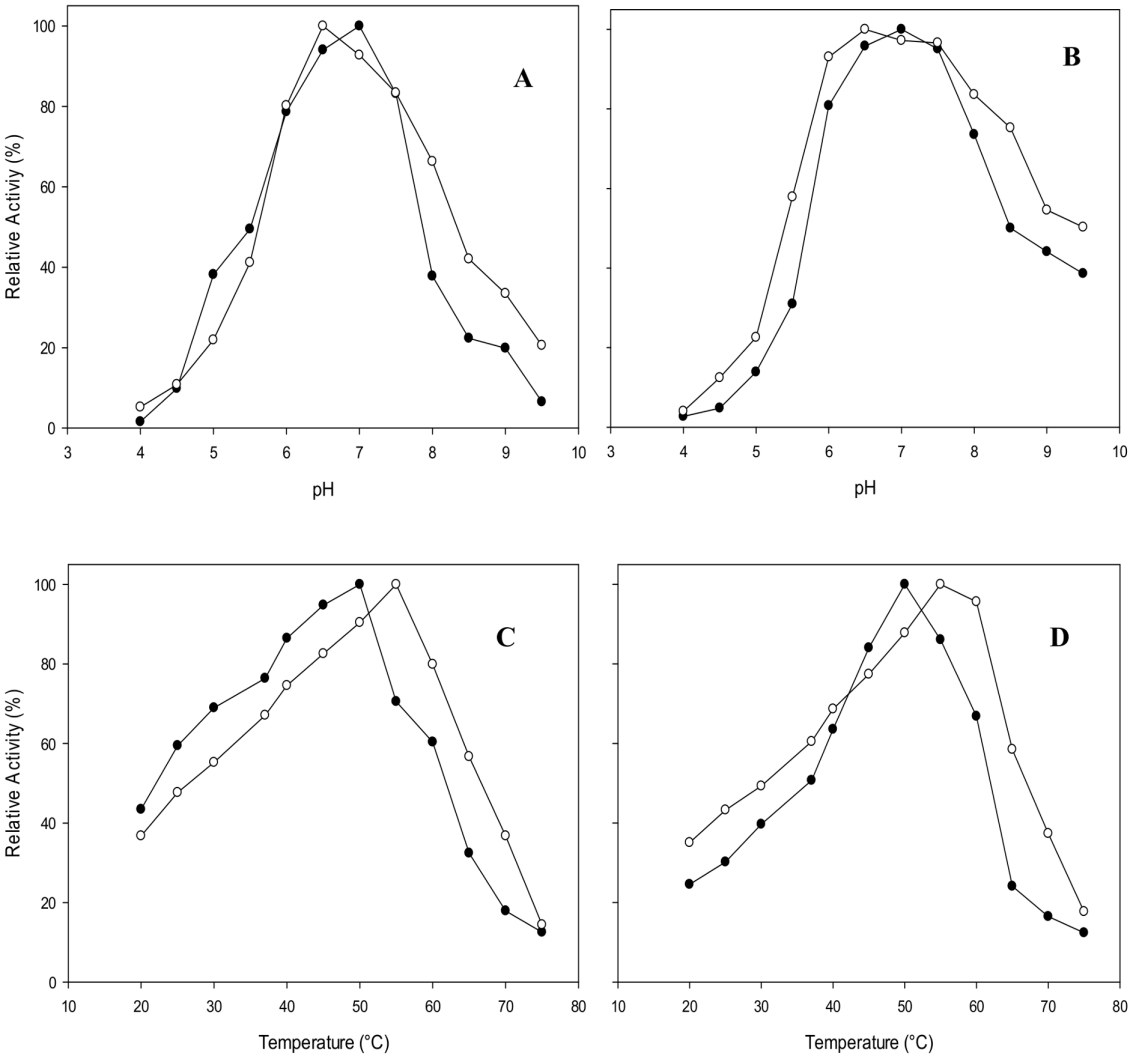
pH (A and B) and temperature (C and D) optimum of β-galactosidase activity for *B. breve* β-gal I (•) and β-gal II (○) using *o*NPG (A and C) and lactose (B and D) as substrate. Values are the mean of two independent experiments and the standard deviation was always less than 5%.

**Figure 2:**
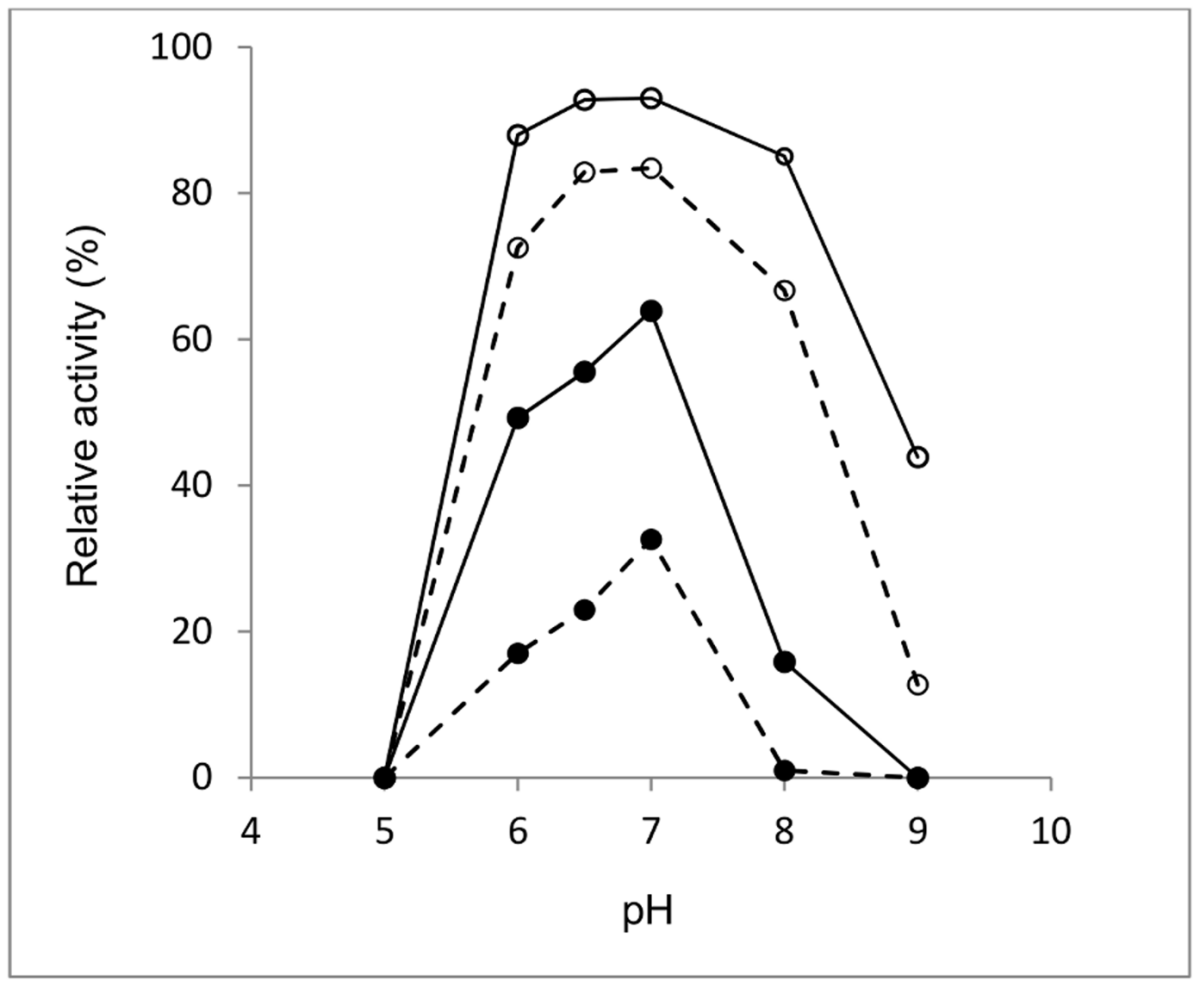
pH stability of the β-galactosidases from *B. breve* β-gal I (•) and β-gal II (○) incubated at 37°C in Britton-Robinson buffer over a pH range of pH 5.0–9.0 for 4 h (solid lines) and 10 h (dashed lines). The residual activity was measured after 4(B) and *o*NPG was used as substrate for the enzyme assay. Values are the mean of two independent experiments and the standard deviation was always less than 5%.

Both *o*NPG and lactose were used as substrates to determine the temperature and pH optimum of β-gal I and β-gal II activity. The pH optimum of β-gal I is pH 7.0 for both *o*NPG and lactose hydrolysis (Figure 1A, B). This enzyme is also most stable at pH 7.0, retaining 60% and approximately 30% of its activity when incubated at pH 7.0 and 37°C for 4 and 10 h, respectively (Figure 2). β-Gal I has a half-life time of activity (τ_1/2_) of 5 h when incubated at pH 7.0 and 37°C. The pH optimum of β-gal II is pH 6.5 for both *o*NPG and lactose hydrolysis (Figure 1A, B), and the enzyme is most stable at pH 6.0–7.0. The residual activities of this enzyme after 10 h of incubation at pH 6.0, 6.5, and 7.0 at 37°C were 72%, 82%, and 83%, respectively (Figure 2). The stability of both enzymes rapidly dropped at pH values below 6.0 or above 8.0.

The optimum temperature of β-gal I activity was 50°C when using both *o*NPG and lactose as substrates under standard assay conditions. In comparison to β-gal I, β-gal II had higher optima, which were at 55°C for both substrates (Figure 1C, D). Both recombinant enzymes showed a half-life time of activity (*τ*_1/2_) of ∼5 months at 4°C when stored in sodium phosphate buffer, pH 6.5. Both enzymes also showed their stability at 30°C with half-life time of activities (*τ*_1/2_) of 73 and 109 h for β-gal I and β-gal II, respectively (Table 3A, B).

**Table 3:** Stability of *β*-galactosidases from *B. breve* at different temperatures in the absence of MgCl_2_ as well as in the presence of 1 and 10 mM MgCl_2_.

(A) β-gal I						
Temperature(°C)	Sodium phosphatebuffer, pH 7		Sodium phosphate buffer, pH 7+1 mM Mg^2+^		Sodium phosphate buffer, pH 7+10 mM Mg^2+^	
	*k*_in_ (h^−1^)	*τ*_1/2_ (h)	*k*_in_ (h^−1^)	*τ*_1/2_ (h)	*k*_in_ (h^−1^)	*τ*_1/2_ (h)
30	1.00 (±0.00)×10^−2^	73	2.00 (±0.01)×10^−3^	428	3.00 (±0.00)×10^−3^	235
37	0.32±0.03	2	2.00 (±0.00)×10^−2^	37	2.30 (±0.00)×10^−2^	28
45	9.56±0.26	0.07	0.96±0.04	0.72	1.18±0.06	0.59
50	36.7±1.2	0.02	9.00±0.39	0.08	10.80±0.37	0.06
**(B) β-gal II**						
**Temperature** **(°C)**	**Sodium phosphate** **buffer, pH 6.5**		**Sodium phosphate buffer, pH 6.5** **+1 mM Mg^2+^**		**Sodium phosphate buffer,** **pH 6.5+10 mM Mg^2+^**	
	***k*** **_in_ (h^−1^)**	***τ*** **_1/2_ (h)**	***k*** **_in_ (h^−1^)**	***τ*** **_1/2_ (h)**	***k*** **_in_ (h^−1^)**	***τ*** **_1/2_ (h)**
30	4.67 (±0.06)×10^−3^	109	2.58 (±0.08)×10^−3^	268	2.34 (±0.16)×10^−3^	297
37	2.07 (±0.07)×10^−2^	33	3.79 (±0.01)×10^−3^	183	3.78 (±0.07)×10^−3^	183
50	5.70±0.04	0.12	0.55±0.01	1.25	0.19±0.0.01	3.7

Data given are the inactivation constants *k*_in_ and the half-life times of activity *τ*_1/2_. Values are the mean of two independent experiments and the standard deviation was always less than 5%.

Thermal stability of both β-galactosidases I and II was significantly improved in the presence of MgCl_2_. Table 3 (A, B) shows the effect of 1 and 10 mM of MgCl_2_ on the thermostability of β-gal I and β-gal II at 37°C and higher. In the presence of 1 mM MgCl_2_ β-gal I showed 19-, 10- and 4-fold increase in *τ*_1/2_ at 37°C, 45°C and 50°C, respectively. A further increase of the Mg^2+^ concentration to 10 mM was less effective with respect to stabilization of this enzyme. At all conditions tested, β-gal II was found to be more stable than β-gal I. In the presence of 1 mM MgCl_2_ *τ*_1/2_ of β-gal II activity at 50°C was increased to 1.25 h, compared to 0.12 h without Mg^2+^. A further increase of the Mg^2+^ concentration to 10 mM was more effective in improving thermostability of β-gal II activity. It should be noted that, while Mg^2+^ increased stability considerably, it had no effect on activity of both β-gal I and β-gal II. Stability was further studied by differential scanning calorimetry (DSC). Both enzymes showed a single endothermic peak in the DSC scans which fitted very well on the basis of a two-state transition model (data not shown). The observed melting temperatures *T*_m_, 49.97 and 55.58°C for β-gal I and β-gal II, B, respectively, are in excellent agreement with the optimum temperatures of these two enzymes as shown in Figure 1C, D.

### Substrate specificity

The two β*-*galactosidases from *B. breve* displayed a narrow substrate range when using chromogenic substances. Both enzymes showed 1% activity (relative to *o*NPG) when using 2-nitrophenyl-β-D-glucopyranoside while no activity (<0.05%) was observed when 4-nitrophenyl-β-D-mannopyranoside, 4-nitrophenyl-α-D-galactopyranoside, or 4-nitrophenyl-α-D-glucopyranoside were used as substrates.

Activities of *B. breve* β-galactosidases with individual galactosides are expressed as relative hydrolysis (or conversion) of each substrate after 30 and 60 min (Table 4). β-Gal I shows high hydrolytic activity towards lactose with more than 99% hydrolysis after 30 minutes of hydrolytic reaction. β*-*D-Gal*p*-(1→6)-D-Glc (allolactose), β*-*D-Gal*p-*(1→3)-D-Lac, β*-*D-Gal*p*-(1→3)-D-Glc and β*-*D-Gal*p-*(1→3)-D-Gal were hydrolyzes at comparable rates (Table 4). β-Gal II also showed high activities with lactose, β*-*D-Gal*p*-(1→6)-D-Glc (allolactose) and β*-*D-Gal*p-*(1→3)-D-Lac but these substrates were hydrolyzed at slightly lower rates than that by β-gal I. Both enzymes show low activity with the disaccharide β*-*D-Gal*p-*(1→4)-D-Gal and the trisaccharides β*-*D-Gal*p-*(1→4)-D-Lac and β*-*D-Gal*p-*(1→6)-D-Lac, which is evident from the slow hydrolysis rates of these substrates.

**Table 4:** Relative hydrolytic activities of *B. breve* β-galactosidases for individual galactosides.

Substrate	% Conversion			
	β-gal I		β-gal II	
	30 min	60 min	30 min	60 min
Lactose	>99	>99	86.3	>99
β*-*D-Gal*p*-(1→6)-D-Glc	>99	>99	98.3	>99
β*-*D-Gal*p*-(1→3)-D-Glc	97.7	>99	61.7	88.6
β*-*D-Gal*p-*(1→3)-D-Gal	98.7	>99	73.1	90.9
β*-*D-Gal*p-*(1→4)-D-Gal	11.6	12.4	4.7	11.6
β*-*D-Gal*p-*(1→6)-D-Gal	52.1	79.0	48.2	80.4
β*-*D-Gal*p-*(1→3)-D-Lac	>99	>99	85.7	97.6
β*-*D-Gal*p-*(1→4)-D-Lac	22.1	29.1	21.8	26.2
β*-*D-Gal*p-*(1→6)-D-Lac	10.3	30.4	5.9	14.9

Results are expressed as a percentage of hydrolysis (or conversion) of each substrate after 30 and 60 min.

### GOS synthesis

**Figure 3:**
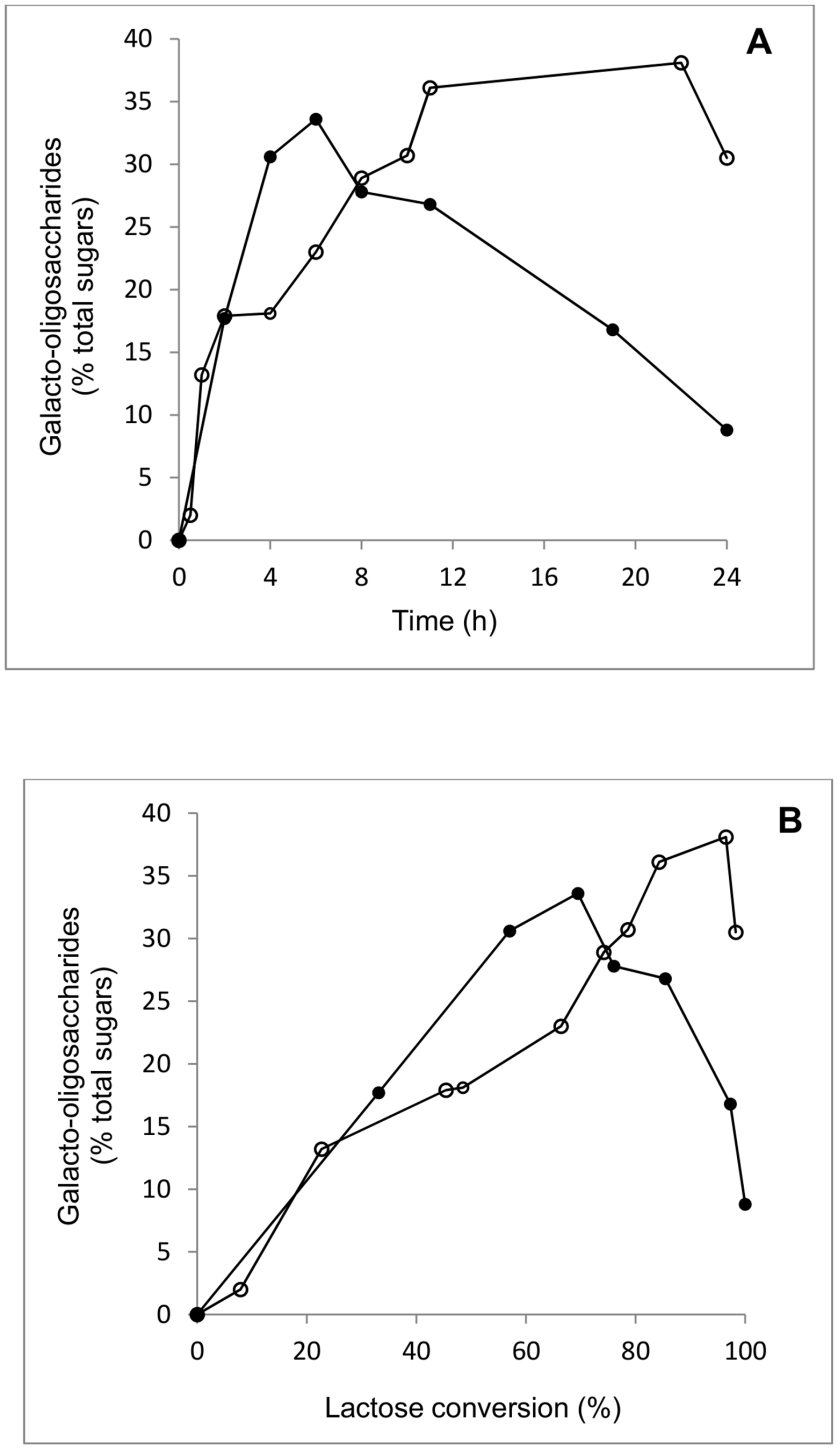
Time course of GOS formation (A) and formation and degradation of GOS during lactose conversion (B) catalyzed by *B. breve* β-gal I (•) and β-gal II (○). The reaction was performed at 30°C at an initial lactose concentration of 200 g L^−1^ in sodium phosphate buffer (pH 6.5) and 1 mM MgCl_2_ using 1.5 U_Lac_ mL^−1^_._ Values are the mean of two independent experiments and the standard deviation was always less than 5%.

Figure 3 shows GOS (including non-lactose disaccharides) formation of a typical discontinuous conversion reaction at 30°C with an initial lactose concentration of 200 g L^−1^ in sodium phosphate buffer (pH 6.5) and 1 mM MgCl_2_ using 1.5 U_Lac_ mL^−1^ of enzyme. Under these conditions, maximum GOS yields of 33% of total sugars (after 6 h of reaction and when 70% of the initial lactose were converted), and 38% of total sugars (after 22 h of reaction at 96% lactose conversion) were obtained with β-gal I and β-gal II, respectively. The amount of GOS expressed as percentage of total sugars is constantly rising from 0 to 33% and from 0 to 38%, when lactose conversion increased up to ∼70% and ∼90% using β-gal I and β-gal II, respectively. After these points, at which maximum GOS yields were obtained, the concentration of GOS decreased because they are also subjected to hydrolysis by the β-galactosidases. This is particularly pronounced for β-gal I. When the concentration of β-gal I in the conversion reaction was reduced to 1.0 U_Lac_ mL^−1^, a slight difference on the maximum GOS yield, which was 30% of total sugars at 70% lactose conversion, was observed. Interestingly, when the concentration of β-gal II in the conversion reaction was increased to 2.5 U_Lac_ mL^−1^, maximum GOS yield increased from 38% to 44% of total sugars, which was obtained at 84% lactose conversion, and also the time needed to obtain this maximum GOS yield was reduced to 6 h (data not shown).

**Figure 4:**
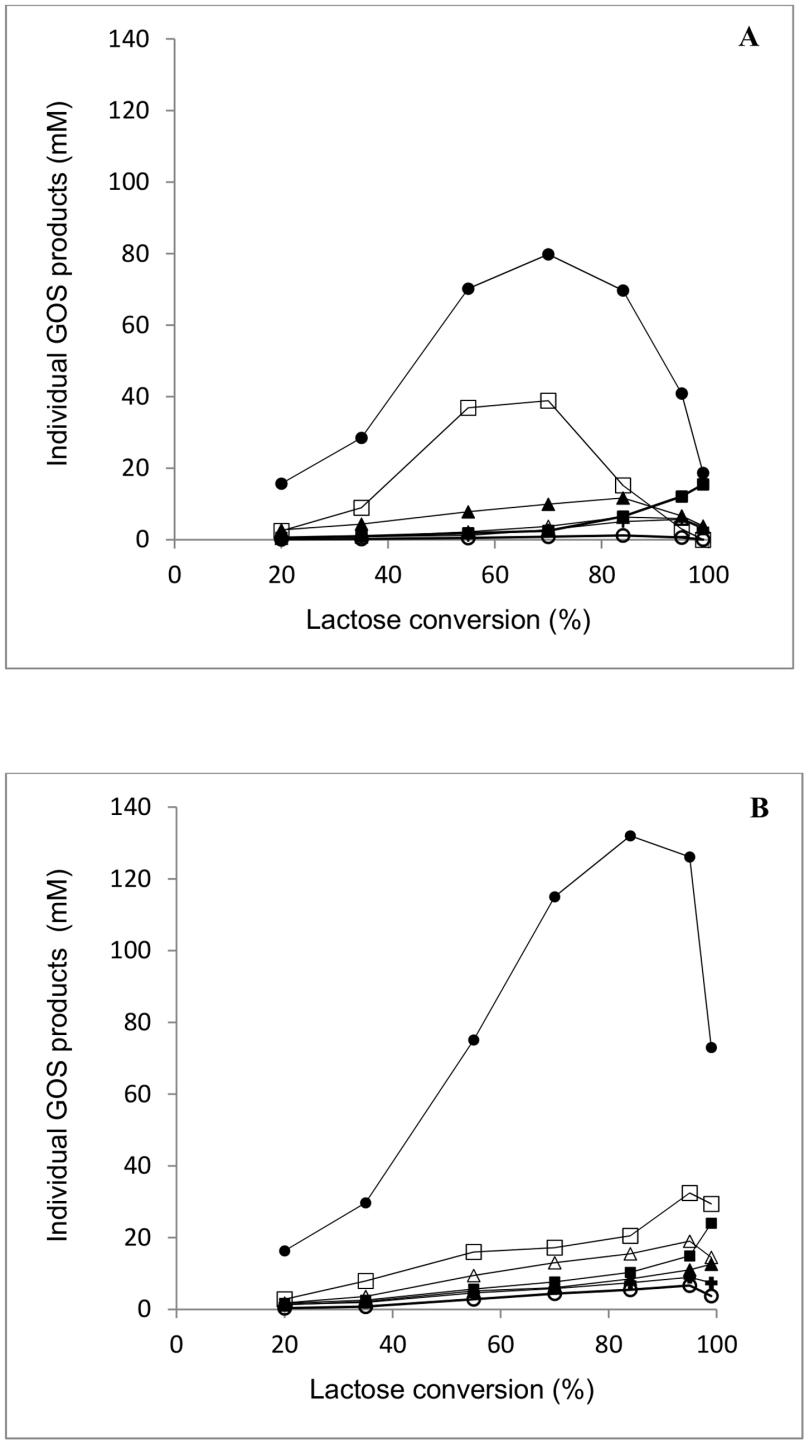
Formation and degradation of individual GOS formed by *B. breve* β-gal I (A) and β-gal II (B) during lactose conversion. Reaction conditions: initial lactose concentration of 200 g L^−1^ in 50 mM sodium phosphate buffer (pH 6.5) with 1 mM Mg^2+^ and 30°C and 1.0 U_Lac_ mL^−1^ β-gal I or 2.5 U_Lac_ mL^−1^ β-gal II. Symbols: (•) D-Gal*p*-(1→6)-D-Glc; (▪) D-Gal*p*-(1→6)-D-Gal; (▴) Gal*p*-(1→3)-D-Gal; (Δ) D-Gal*p*-(1→3)-D-Glc; (□) D-Gal*p*-(1→3)-D-Lac; (○) D-Gal*p*-(1→4)-Lac, (+) D-Gal*p*-(1→6)-D-Lac.

Individual GOS can be separated effectively using a Carbopac PA1 column for HPAEC with pulsed amperometric detection (Figure S2A, B). It was possible to identify the main products of transgalactosylation by both β-gal I and β-gal II. These main transferase products formed and degraded at different lactose conversion are presented in Table S1 and Figure 4A, B. The predominant oligosaccharide product was identified as β*-*D-Gal*p*-(1→6)-D-Glc (allolactose), accounting for approximately 45% and 50% of the GOS formed by transgalactosylation by β-gal I and β-gal II, respectively, at maximum total GOS yield. β*-*D-Gal*p-*(1→3)-D-Lac was identified as the second important transferase product at the maximum total GOS yield point, contributing approximately 32% and 16% of the total GOS formed by β-gal I and β-gal II, respectively. Other identified products, including β-d-Gal*p*-(1→3)-Glc, β-d-Gal*p*-(1→3)-Gal, β-d-Gal*p*-(1→6)-Gal, β-d-Gal*p*-(1→6)-Lac and β-d-Gal*p*-(1→4)-Lac, make up approximately 12% and 20% of total GOS (at total GOS maximum yield) formed using β-gal I and β-gal II, respectively. 4′-Galactobiose was not detected at all during the course of lactose conversion. It should be noted that the unidentified peaks 8 and 14 were present in detectable concentrations (Figure S2A, B). However, the structure of these components has yet to be determined.

## Discussion

In this study, two GH2 β-galactosidases, β-gal I and β-gal II, of the LacZ type of *B. breve* DSM 20213 were cloned, heterologously expressed in *E. coli* and biochemically characterized. The activity yields of 683 and 169 kU L^−1^ obtained in simple shaken flask cultures for β-gal I and β-gal II, respectively, correspond to values of ∼1.5 and 0.86 g of recombinant protein produced per L of medium. These yields are significantly higher than the recently reported maximum yield of a β-galactosidase (BbgIV) from *Bifidobacterium bifidum* NCIMB 41171 expressed in *E. coli* DH5α, which was 0.4–0.5 g BbgIV per L of culture medium [41]. Furthermore, ∼31% and 18% of the total soluble protein in the cellular extracts of *E. coli* overexpressing the genes encoding β-gal I and β-gal II, respectively, can be attributed to the recombinant proteins as judged by the specific activities. Co-expression of the chaperones GroEL/GroES significantly boosted expression levels of both β-galactosidases.

Kinetic constants were determined for the two substrates lactose and *o*NPG. The *K_m_* values determined for lactose, 15.3 and 7.5 mM for β-gal I and β-gal II, respectively, are lower compared to the values reported for other β-galactosidases from *Bifidobacterium* spp. including *B. adolescentis* β-gal II (60 mM) [22], *B. breve* B24 (95.6 mM) [34], *B. bifidum* β-gal I (29.9 mM) and β-gal II (47.1 mM) [25], as well as fungal and yeast β-galactosidases that are commonly employed in technological applications, for example *A. oryzae* (36–180 mM), *A. niger* (54–99 mM), *K. fragilis* (15–52 mM) [42], *K. lactis* (35 mM) [43]. These *K*_m_ values of *B. breve β*-galactosidases compare favorably with the values reported for *β*-galactosidases from *B. bifidum β*-gal III (9.56 mM) [25], *L. reuteri* (13 mM) [26], and *L. crispatus* (14 mM) [44]. These relatively low *K*_m_ values of the *B. breve* β-galactosidases can be an advantage, e.g. when the complete hydrolysis of lactose is desired.

The inhibition by the end product galactose is moderate as is evident from the ratio of *K*_i_ to *K*_m_ calculated for this competitive inhibitor. This ratio of *K*_i_ to *K*_m_ represents a specificity constant, which determines preferential binding of the substrate lactose versus that of the monosaccharide end product, hence a high value for this ratio is desirable for efficient hydrolysis of lactose. The *B. breve* β-galactosidases display values for the *K*_i,Gal_/*K*_m,Lac_ ratio of 1.8 and 3.6 for β-gal I and β-gal II, respectively, indicating low inhibition. Values for the *K*_i,Gal_/*K*_m,Lac_ ratio reported for example for *B. licheniformis*, *A. oryzae, A. niger* and *K. fragilis* β-galactosidases are as low as 0.0055, 0.01, 0.006 and 0.84, respectively, indicating severe inhibition by the end product galactose [45], [46].

Recombinant GH2 β-galactosidases from the infant isolate *B. breve* were found to be suitable for the production of GOS via transgalactosylation. Maximum total GOS yields of 33% and 44% were obtained when β-gal I and β-gal II were used in discontinuous conversion reactions with an initial lactose concentration of 200 g L^−1^. The conversions were performed with this initial lactose concentration based on the solubility of lactose at ambient temperature. An increase in reaction temperature would help to increase the solubility of lactose, however this was not possible since both enzymes lack sufficient stability above 30°C. The maximum GOS yield obtained with β-gal II is comparable to the reported yields obtained with other β-galactosidases from *Bifidobacterium* spp., for example *B. angulatum* (43.8%), *B. bifidum* BB-12 (37.6%), *B. adolescentis* (43.1%) [47], and *B. breve* B24 (42%) [34], however the lactose conversions for GOS synthesis using these β-galactosidases were performed with initial lactose concentration of 30% (w/w). Additionally, Goulas et al. [48] reported a yield of 47% of GOS using BbgIV from *B. bifidum* NCIMB41171 at 40°C and 40% (w/w) initial lactose concentration, while Osman et al. [49] obtained a yield of 55% at 65°C and 43% (w/w) initial lactose concentration using the same enzyme. It was found by many authors that the initial lactose concentration has a significant impact on GOS yields [16], [27], [50].

Both enzymes show highest hydrolytic affinities towards lactose, allolactose and β*-*D-Gal*p-*(1→3)-D-Lac among the substrates tested. It is conceivable that the ‘probiotic’ β-galactosidases, which rapidly *hydrolyze* certain galacto-oligosaccharide structures, can preferentially *form* these glycosidic linkages as well when acting in transgalactosylation mode, and this is again confirmed in this study. The predominant transgalactosylation products were identified as β*-*D-Gal*p*-(1→6)-D-Glc (allolactose) and β*-*D-Gal*p-*(1→3)-D-Lac, together accounting for more than 75% and 65% of the GOS formed by transgalactosylation by β-gal I and β-gal II, respectively. Both enzymes show very low activity towards β*-*D-Gal*p-*(1→4)-D-Gal, and interestingly, this disaccharide was not detected and hence formed at all during lactose conversion by β-gal I and β-gal II.

**Figure 5:**
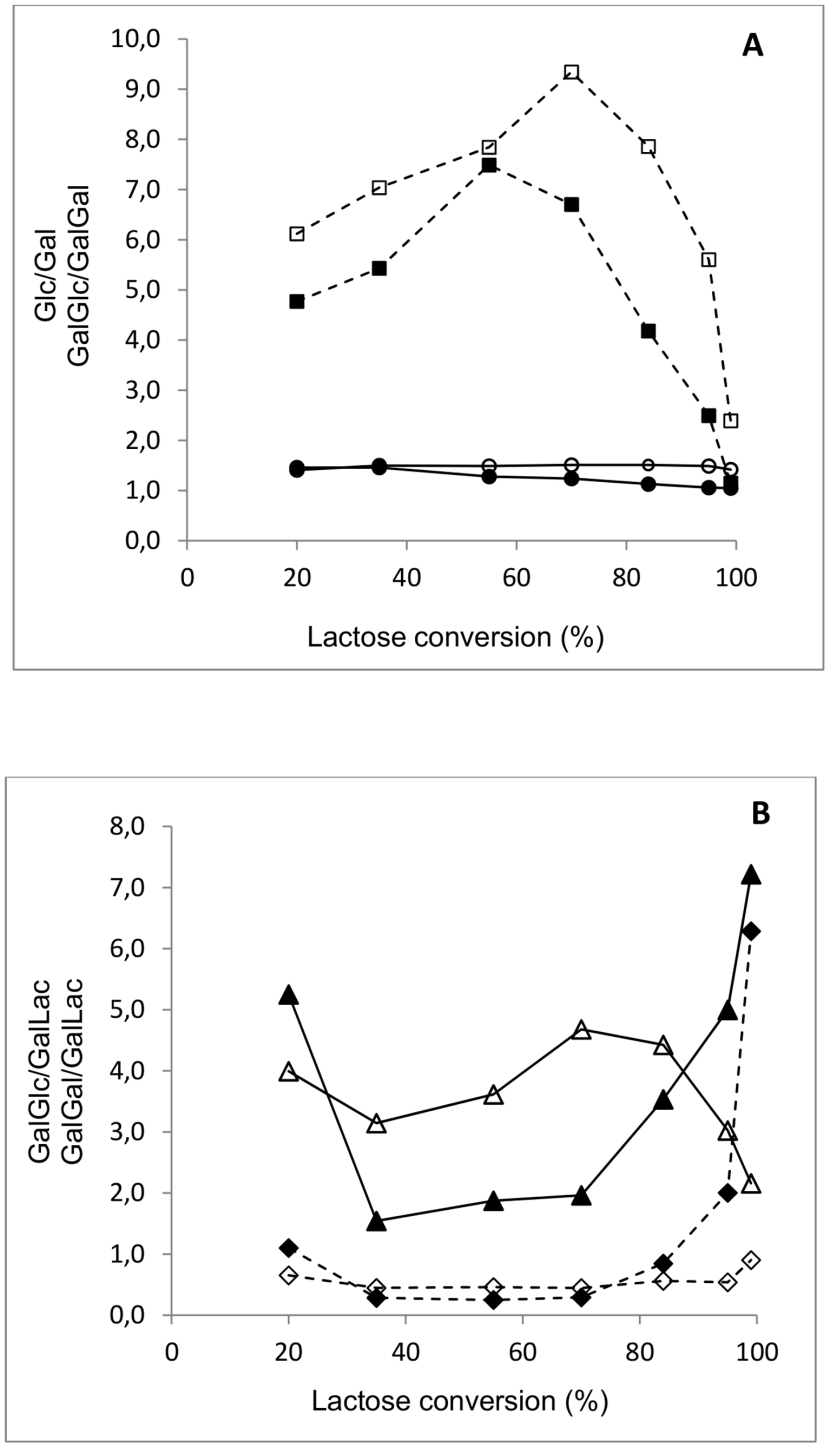
D-Glucose/D-Galactose (solid lines) and GalGlc/GalGal (dashed lines) ratios (A); GalGlc/GalLac (solid lines) and GalGal/GalLac (dashed lines) ratios (B) during lactose conversion by *B. breve* β-gal I (close symbol) and β-gal II (open symbol). The reactions were performed at 30°C at an initial lactose concentration of 200 g L^−1^ in sodium phosphate buffer (pH 6.5) and 1 mM MgCl_2_ with 1.0 U_Lac_ mL^−1^ β-gal I or 2.5 U_Lac_ mL^−1^ β-gal II.

Transgalactosylation is described to involve intermolecular as well as intramolecular reactions. Intramolecular or direct galactosyl transfer to D-glucose yields regio-isomers of lactose, and disaccharides are formed right from the beginning of the reaction even when hardly any monosaccharide galactosyl acceptors are available. In this reaction pathway the noncovalently enzyme-bound glucose is not released from the active site but linked immediately to the galactosyl enzyme intermediate. Different transfer rates for different acceptors are to some extent responsible for these phenomena. Figure 5A reveals the ratio between GalGlc and GalGal disaccharides at all lactose conversion levels formed during transgalactosylation using β-gal I and β-gal II. This ratio was as high as ≈5 (for β-gal I) or ≈6 (β-gal II) during the initial phase of the reaction (at 20% lactose conversion), at which the concentration of the main hydrolysis products D-Glc and D-Gal are relatively low. This indicates that D-glucose is an excellent galactosyl acceptor, in fact it is a far better acceptor than D-galactose for galactosyl transfer by both of these two enzymes. Figure 5B shows that D-glucose is also a better galactosyl acceptor than D-lactose when looking at the ratio between GalGlc disaccharides and GalLac trisaccharides. Especially at the beginning of the reaction this ratio was ≈5 (for β-gal I) or ≈4 (β-gal II) at 20% lactose conversion. This indicates that at least D-Gal*p*-(1→6)-D-Glc is formed by intramolecular transgalactosylation, that is, the D-Gal moiety is transferred onto D-Glc before it can leave the active site of β-galactosidase and another acceptor molecule or water can enter the active site. Since both enzymes form β-d-Gal*p*-(1→4)-Lac, it is conceivable that the galactosyl moiety can also be transferred onto glucose to form lactose (β-d-Gal*p*-(1→4)-Glc) as an intramolecular transgalactosylation product; however, this cannot be distinguished experimentally from lactose provided as a substrate. As β*-*D-Gal*p-*(1→3)-D-Lac is the second main product during transgalactosylation after D-Gal*p*-(1→6)-D-Glc and when looking at the ratio between GalGal disaccharides and GalLac trisaccharides (figure 5B), it can be concluded that D-lactose is preferred to D-galactose as galactosyl acceptor during intermolecular transgalactosylation.

In conclusion, two GH2 β-galactosidases from *B. breve* DSM 20213, β-gal I and β-gal II, were studied in detail regarding their biochemical properties, distribution of oligosaccharides formed, and linkages preferentially synthesized in transgalactosylation mode. Both enzymes were found to be very well suited for the production of galacto-oligosaccharides, components that are of great interest because of their use in functional food. The resulting GOS mixtures contained relatively high fractions of allolactose, which results from the fact that glucose is a far better acceptor for galactosyl transfer than galactose and lactose, and intramolecular transgalactosylation contributes significantly to the formation of this disaccharide. 3′-Galactosyl-lactose was found to be the major trisaccharide in the GOS mixtures. The β-galactosidases from *B. breve* DSM 20213 should be of considerable interest for the production of prebiotic GOS.

## Supporting Information

Figure S1 **SDS-PAGE analysis of recombinant β** ***-*** **galactosidases from** ***B. breve*** **stained with Coomassie blue.** Lanes 1 and 4 shows the molecular mass marker (Amersham); lanes 2 and 5 are the crude extracts of β*-*gal I and β*-*gal II, lanes 3 and 6 are the purified enzymes of β-gal I and β-gal II.
(DOCX)

Figure S2 **Separation and quantification by HPAEC-PAD of individual GOS produced during lactose conversion catalyzed by** ***B. breve*** **β-gal I (A), and** ***B. breve*** **β-gal II (B).** The identified compounds are (1) D-galactose, (2) D-glucose, (3) D-Galp-(1→6)-D-Gal, (4) D-Galp-(1→6)-D-Glc (allolactose), (5) D-Galp-(1→4)-D-Glc (lactose), (6) D-Galp-(1→3)-D-Gal, (7) D-Galp-(1→6)-Lac, (9) D-Galp-(1→3)-D-Glc, (13) D-Galp-(1→4)-Lac and (15) D-Galp-(1→3)-Lac. Peaks 8, 10–12, 14, and 16–20 were not identified.
(DOCX)

Table S1 **Individual GOS components produced by the transgalactosylation reaction of β-gal I (I) and β-gal II (II) from** ***Bifidobacterium breve*** **DSM 20031 using lactose as substrate.** The reaction was performed at 30°C at an initial lactose concentration of 200 g L^−1^ in sodium phosphate buffer (pH 6.5) and 1 mM MgCl_2_ using 1.0 U_Lac_ mL^−1^ (β-gal I) or 2.5 U_Lac_ mL^−1^ (β-gal II).
(DOCX)
