# Supplementary figures and images for: Two β-Galactosidases from the Human Isolate Bifidobacterium breve DSM 20213: Molecular Cloning and Expression, Biochemical Characterization and Synthesis of Galacto-Oligosaccharides
Source: PLoS One. 2014 Aug 4;9(8):e104056. doi: 10.1371/journal.pone.0104056 (PMC4121272; doi:10.1371/journal.pone.0104056)

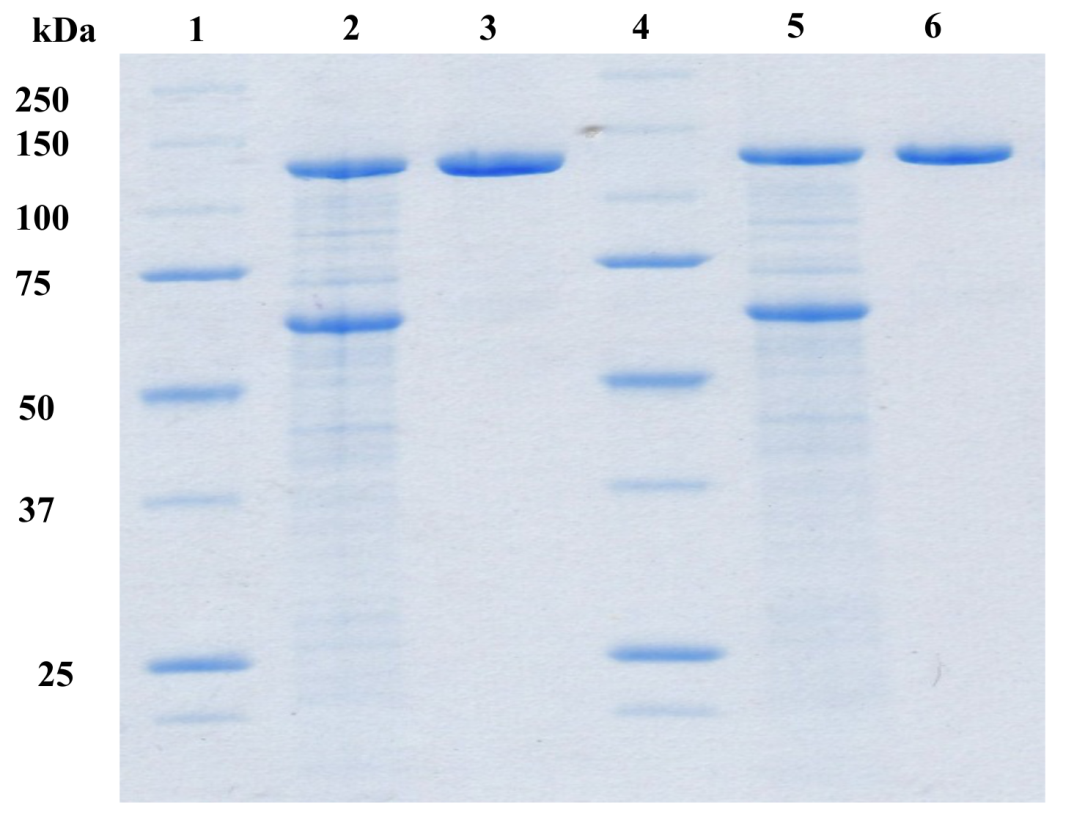


Figure S1.

Supplement: Figure S1 — SDS-PAGE analysis of recombinant β - galactosidases from B. breve stained with Coomassie blue. Lanes 1 and 4 shows the molecular mass marker (Amersham); lanes 2 and 5 are the crude extracts of β-gal I and β-gal II, lanes 3 and 6 are the purified enzymes of β-gal I and β-gal II. (DOCX) [file pone.0104056.s001.docx]

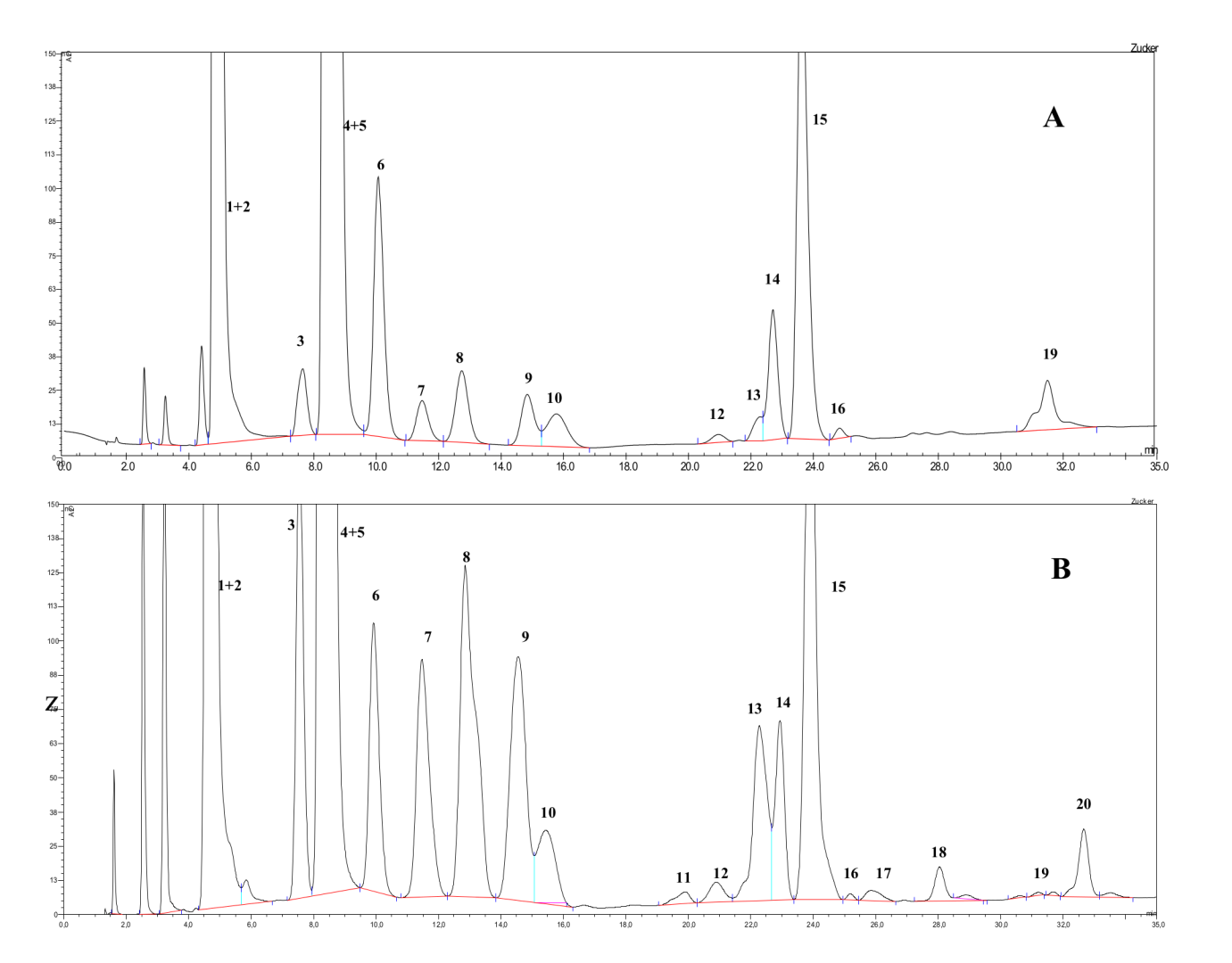


Figure S2.

Supplement: Figure S2 — Separation and quantification by HPAEC-PAD of individual GOS produced during lactose conversion catalyzed by B. breve β-gal I (A), and B. breve β-gal II (B). The identified compounds are (1) D-galactose, (2) D-glucose, (3) D-Galp-(1→6)-D-Gal, (4) D-Galp-(1→6)-D-Glc (allolactose), (5) D-Galp-(1→4)-D-Glc (lactose), (6) D-Galp-(1→3)-D-Gal, (7) D-Galp-(1→6)-Lac, (9) D-Galp-(1→3)-D-Glc, (13) D-Galp-(1→4)-Lac and (15) D-Galp-(1→3)-Lac. Peaks 8, 10–12, 14, and 16–20 were not identified. (DOCX) [file pone.0104056.s002.docx]
