# Supplementary material for: Two β-Galactosidases from the Human Isolate Bifidobacterium breve DSM 20213: Molecular Cloning and Expression, Biochemical Characterization and Synthesis of Galacto-Oligosaccharides
Source: PLoS One. 2014 Aug 4;9(8):e104056. doi: 10.1371/journal.pone.0104056 (PMC4121272; doi:10.1371/journal.pone.0104056)
Supplement: Table S1 — Individual GOS components produced by the transgalactosylation reaction of β-gal I (I) and β-gal II (II) from Bifidobacterium breve DSM 20031 using lactose as substrate. The reaction was performed at 30°C at an initial lactose concentration of 200 g L−1 in sodium phosphate buffer (pH 6.5) and 1 mM MgCl2 using 1.0 ULac mL−1 (β-gal I) or 2.5 ULac mL−1 (β-gal II). (DOCX) [file pone.0104056.s003.docx]

| **Table S1**. Individual GOS components produced by the transgalactosylation reaction of β-gal I (I) and β-gal II (II) from *Bifidobacterium breve* DSM 20031 using lactose as substrate. The reaction was performed at 30°C at an initial lactose concentration of 200 g L^-1^ in sodium phosphate buffer (pH 6.5) and 1 mM MgCl_2_ using 1.0 U_Lac_ mL^-1^ (β-gal I) or 2.5 U_Lac_ mL^-1^ (β-gal II) | | | | | | | | | | | | | | | | | | | | | | | | | |  |
| --- | --- | --- | --- | --- | --- | --- | --- | --- | --- | --- | --- | --- | --- | --- | --- | --- | --- | --- | --- | --- | --- | --- | --- | --- | --- | --- |
| GOS components^a^ | Degree of Lactose Conversion | | | | | | | | | | | | | | | | | | | | | | | | |  |
|  | 20% | |  | 35% | |  | 55% | | |  | 70% | | | |  | | 84% | |  | 95% | |  | 99% | | |  |
|  | I | II |  | I | II |  | I | | II |  | I | | II | |  | | I | II |  | I | II |  | I | II | |  |
| GOS components (g L^-1^) |  |  |  |  |  |  |  | |  |  |  | | | |  | |  |  |  |  |  |  |  |  | |  |
| D-Gal*p*-(1🡪6)-D-Glc | 5.39 | 5.59 |  | 9.75 | 10.2 |  | 24.0 | 25.7 | |  | 27.3 | 39.2 | |  | | 23.8 | | 45.3 |  | 14.0 | 43.2 |  | 6.39 | | 25.0 | |
| D-Gal*p*-(1🡪6)-D-Gal | 0.21 | 0.58 |  | 0.35 | 0.88 |  | 0.63 | 1.94 | |  | 0.86 | 2.62 | |  | | 2.22 | | 3.52 |  | 4.13 | 5.11 |  | 5.30 | | 8.20 | |
| D-Gal*p*-(1🡪3)-D-Gal | 0.95 | 0.43 |  | 1.50 | 0.74 |  | 2.68 | 1.75 | |  | 3.41 | 2.07 | |  | | 4.01 | | 2.90 |  | 2.28 | 3.77 |  | 1.32 | | 4.30 | |
| D-Gal*p*-(1🡪3)-D-Glc | 0.16 | 0.60 |  | 0.28 | 1.23 |  | 0.76 | 3.23 | |  | 1.29 | 4.45 | |  | | 2.17 | | 5.30 |  | 2.03 | 6.51 |  | 1.21 | | 4.95 | |
| D-Gal*p*-(1🡪3)-Lac | 1.25 | 1.42 |  | 9.04 | 3.99 |  | 18.6 | 8.08 | |  | 19.6 | 10.4 | |  | | 7.64 | | 14.3 |  | 1.55 | 16.4 |  | <0.01 | | 14.9 | |
| D-Gal*p*-(1🡪4)-Lac | 0.08 | 0.16 |  | 0.12 | 0.38 |  | 0.26 | 1.42 | |  | 0.44 | 2.21 | |  | | 0.62 | | 2.75 |  | 0.33 | 3.35 |  | 0.04 | | 1.88 | |
| D-Gal*p*-(1🡪6)-Lac | 0.22 | 0.69 |  | 0.44 | 0.97 |  | 0.61 | 2.30 | |  | 1.42 | 2.92 | |  | | 2.57 | | 3.73 |  | 2.88 | 4.50 |  | 1.51 | | 3.74 | |
| Other GOS | 0.00 | 0.00 |  | 7.89 | 0.49 |  | 4.58 | 3.48 | |  | 6.34 | 2.24 | |  | | 9.20 | | 9.96 |  | 0.0 | 2.90 |  | 0.00 | | 9.25 | |
| Total GOS | 8.26 | 9.47 |  | 29.4 | 18.85 |  | 52.2 | 47.9 | |  | 60.7 | 66.1 | |  | | 44.6 | | 87.8 |  | 27.2 | 85.7 |  | 15.8 | | 72.2 | |
| GOS components |  |  |  |  |  |  |  |  | |  |  |  | |  | |  | |  |  |  |  |  |  | |  | |
| D-Gal*p*-(1🡪6)-D-Glc | 15.7 | 16.3 |  | 28.5 | 29.7 |  | 70.2 | 75.1 | |  | 79.8 | 115 | |  | | 69.7 | | 132 |  | 40.9 | 126.1 |  | 18.7 | | 73.0 | |
| D-Gal*p*-(1🡪6)-D-Gal | 0.61 | 1.69 |  | 1.02 | 2.57 |  | 1.84 | 5.67 | |  | 2.51 | 7.65 | |  | | 6.49 | | 10.3 |  | 12.1 | 14.9 |  | 15.5 | | 24.0 | |
| D-Gal*p*-(1🡪3)-D-Gal | 2.78 | 1.26 |  | 4.38 | 2.16 |  | 7.83 | 5.11 | |  | 9.96 | 6.05 | |  | | 11.7 | | 8.47 |  | 6.67 | 11.0 |  | 3.86 | | 12.6 | |
| D-Gal*p*-(1🡪3)-D-Glc | 0.47 | 1.75 |  | 0.82 | 3.59 |  | 2.22 | 9.44 | |  | 3.77 | 13.0 | |  | | 6.34 | | 15.5 |  | 5.93 | 19.0 |  | 3.53 | | 14.5 | |
| D-Gal*p*-(1🡪3)-Lac | 2.48 | 2.82 |  | 17.9 | 7.91 |  | 36.9 | 16.0 | |  | 38.9 | 20.7 | |  | | 15.2 | | 20.5 |  | 3.02 | 32.43 |  | 0.0 | | 29.4 | |
| D-Gal*p*-(1🡪4)-Lac | 0.16 | 0.32 |  | 0.24 | 0.75 |  | 0.52 | 2.82 | |  | 0.87 | 4.38 | |  | | 1.23 | | 5.45 |  | 0.65 | 6.64 |  | 0.08 | | 3.73 | |
| D-Gal*p*-(1🡪6)-Lac | 0.44 | 1.38 |  | 0.87 | 1.93 |  | 1.22 | 4.57 | |  | 2.81 | 5.78 | |  | | 5.09 | | 7.39 |  | 5.70 | 8.92 |  | 3.00 | | 7.41 | |
| GOS components (% mass of total GOS) |  |  |  |  |  |  |  |  | |  |  |  | |  | |  | |  |  |  |  |  |  | |  | |
| D-Gal*p*-(1🡪6)-D-Glc | 65.3 | 59.0 |  | 33.2 | 54.0 |  | 46.1 | 53.7 | |  | 45.1 | 59.3 | |  | | 53.4 | | 51.6 |  | 51.5 | 50.4 |  | 40.5 | | 34.6 | |
| D-Gal*p*-(1🡪6)-D-Gal | 2.54 | 6.12 |  | 1.19 | 4.67 |  | 1.21 | 4.05 | |  | 1.42 | 3.96 | |  | | 4.97 | | 4.01 |  | 15.2 | 5.97 |  | 33.6 | | 11.4 | |
| D-Gal*p*-(1🡪3)-D-Gal | 11.5 | 4.54 |  | 5.11 | 3.93 |  | 5.14 | 3.65 | |  | 5.62 | 3.13 | |  | | 8.98 | | 3.30 |  | 8.39 | 4.40 |  | 8.37 | | 5.95 | |
| D-Gal*p*-(1🡪3)-D-Glc | 1.94 | 6.33 |  | 0.95 | 6.53 |  | 1.46 | 6.74 | |  | 2.13 | 6.73 | |  | | 4.87 | | 6.03 |  | 7.47 | 7.60 |  | 7.67 | | 6.85 | |
| D-Gal*p*-(1🡪3)-Lac | 15.1 | 15.0 |  | 30.8 | 21.2 |  | 35.7 | 16.8 | |  | 32.3 | 15.8 | |  | | 17.1 | | 16.3 |  | 5.70 | 19.1 |  | 0.0 | | 20.6 | |
| D-Gal*p*-(1🡪4)-Lac | 0.97 | 1.69 |  | 0.41 | 2.02 |  | 0.50 | 2.96 | |  | 0.73 | 3.34 | |  | | 1.39 | | 3.13 |  | 1.21 | 3.91 |  | 0.25 | | 2.60 | |
| D-Gal*p*-(1🡪6)-Lac | 2.66 | 7.33 |  | 1.49 | 5.17 |  | 1.18 | 4.81 | |  | 2.34 | 4.41 | |  | | 5.76 | | 4.24 |  | 10.6 | 5.25 |  | 9.60 | | 5.17 | |
| ^a^ D-Gal*p*-(1🡪4)-D-Gal was not detected at all lactose conversion level (limit of detection = 0.01 g L^-1^ ). | | | | | | | | | | | | | | | | | | | | | | |  |  | |  |
